# Transcriptional Network Architecture of Breast Cancer Molecular Subtypes

**DOI:** 10.3389/fphys.2016.00568

**Published:** 2016-11-22

**Authors:** Guillermo de Anda-Jáuregui, Tadeo E. Velázquez-Caldelas, Jesús Espinal-Enríquez, Enrique Hernández-Lemus

**Affiliations:** ^1^Computational Genomics, National Institute of Genomic MedicineMexico City, Mexico; ^2^Complejidad en Biología de Sistemas, Centro de Ciencias de la Complejidad, Universidad Nacional Autónoma de MéxicoMexico City, Mexico

**Keywords:** gene regulatory networks, breast cancer, molecular subtypes, network topology, clinical genomics

## Abstract

Breast cancer heterogeneity is evident at the clinical, histological and molecular level. High throughput technologies allowed the identification of intrinsic subtypes that capture transcriptional differences among tumors. A remaining question is whether said differences are associated to a particular transcriptional program which involves different connections between the same molecules. In other words, whether particular transcriptional network architectures can be linked to specific phenotypes. In this work we infer, construct and analyze transcriptional networks from whole-genome gene expression microarrays, by using an information theory approach. We use 493 samples of primary breast cancer tissue classified in four molecular subtypes: Luminal A, Luminal B, Basal and HER2-enriched. For comparison, a network for non-tumoral mammary tissue (61 samples) is also inferred and analyzed. Transcriptional networks present particular architectures in each breast cancer subtype as well as in the non-tumor breast tissue. We find substantial differences between the non-tumor network and those networks inferred from cancer samples, in both structure and gene composition. More importantly, we find specific network architectural features associated to each breast cancer subtype. Based on breast cancer networks' centrality, we identify genes previously associated to the disease, either, generally (i.e., CNR2) or to a particular subtype (such as LCK). Similarly, we identify LUZP4, a gene barely explored in breast cancer, playing a role in transcriptional networks with subtype-specific relevance. With this approach we observe architectural differences between cancer and non-cancer at network level, as well as differences between cancer subtype networks which might be associated with breast cancer heterogeneity. The centrality measures of these networks allow us to identify genes with potential biomedical implications to breast cancer.

## Background

Breast cancer is a heterogeneous disease. The identification of *molecular subtypes* (Perou et al., 2000) was a major breakthrough in order to categorize this heterogeneity, made possible by the emergence of whole-genome microarray technology (Rueda, 2014). Molecular subtypes associate expression of certain markers to phenotypical differences in cancer, pointing to different prognosis as well as distinct therapies (Liu et al., 2014).

The identification of breast cancer molecular subtypes is a paramount example of the impact of high throughput technologies in the study of cancer. These technologies are able to provide even deeper biological understanding, when analyzed by an integrative, systemic framework. In this regard, *network theory* has emerged as a major tool to achieve this goal (Newman, 2010; Chen et al., 2015) under a systems biology view of matters.

A network is a mathematical construct composed by a set of *nodes* or vertices, and a set of *links* that represent a relation between them. A *biological network*, is a network where nodes represent any kind of biological molecules: genes, transcripts, proteins, metabolites, etc., and links represent physical or chemical interactions between those molecules (Jeong et al., 2000; Hasty et al., 2001; Jeong et al., 2001; Thattai and Van Oudenaarden, 2001; Lee et al., 2002; Maslov and Sneppen, 2002; Davidson and Levin, 2005; Guimera and Amaral, 2005; Levine and Davidson, 2005; Davidson and Erwin, 2006). With gene expression microarray technologies, it is factible to construct **transcriptional networks** where nodes are transcribed genes, and links represent a correlation between expression values of said genes, which point to a *possible* interaction between them at the transcriptional level (Tovar et al., 2015).

Many correlation measures have been implemented in order to construct biologically meaningful transcriptional interaction networks based on the inference of statistical dependency (Friedman et al., 2000; Gardner et al., 2003; Giuliani et al., 2004; Wang et al., 2005; Cowell, 2006; Nielsen and Jensen, 2009). This is especially fitting in view of the isomorphism existing between a network structure and a correlation matrix whose elements are the strength of the interaction between the intervening nodes. We must notice, however, that this correlation structure is usually given in the presence of an accompanying variance structure among gene expression levels (Giuliani et al., 2004). It has long been proven that the best estimator of statistic dependency is *mutual information (MI)* (Basso et al., 2005; Margolin et al., 2006; Hernández-Lemus and Rangel-Escareño, 2011; de Matos Simoes and Emmert-Streib, 2012; Hernández-Lemus and Siqueiros-García, 2013). These statistically inferred networks provide a deeper level of biological understanding in two main directions: giving support to previously identified biological observations, and giving new insights regarding novel biological interactions.

Network structural properties have been related to features in the biological context (Kitano, 2002; Albert, 2005; Serrano, 2007; Hakes et al., 2008). Therefore, analyzing these properties in transcriptional networks may provides us better understanding of the underlying biological phenomena. Global network metrics often provide information regarding the system as a whole; while local parameters provide information regarding the relevance of particular nodes (Barabasi and Oltvai, 2004; Newman, 2010; Barabási et al., 2011; Biane et al., 2016; Robinson and Nielsen, 2016). The transcriptional network approach has proven be useful to unveil transcriptional regulation in cancer (Carro et al., 2010; House et al., 2010; Pe'er and Hacohen, 2011; Madhamshettiwar et al., 2012) and in particular in breast cancer (Van De Vijver et al., 2002; Lim et al., 2009; Cicatiello et al., 2010; Gu et al., 2010; Tovar et al., 2015; Castro et al., 2016).

Transcriptional networks are representations of the regulatory programs behind phenotypes. Given the intrinsic heterogeneity of breast cancer molecular subtypes, a fundamental question which remains unsolved is whether the transcriptional architecture of these subtypes is different. To answer this we constructed transcriptional networks for breast cancer molecular subtypes based on mutual information of genome-wide gene expression. We compared them to a network of healthy mammary tissue.

We identified differences in network architecture between phenotypes. We observed major differences between the cancer subtype networks and the non-tumor network. Particular architectural features were associated to the different molecular subtypes. We find that in these networks, the connectivity of particular genes may indicate differences of their role in the transcriptional program of each subtype. Identifying such differences may be key to understand how the specific transcriptional program shapes a particular phenotype. This in turn, will enhance our insight on the nature of molecular subtypes, with basic and clinical implications.

## Methods

### Sample data and classification

Four hundred and ninty three-microarray expression profiles for breast cancer samples and 61 microarray expression profiles corresponding to healthy breast tissue were collected from several experimental datasets that are available on the Gene Expression Omnibus site (Edgar et al., 2002). We used microarray data from GSE 4922 (Ivshina et al., 2006), 1456 (Pawitan et al., 2005), 7390 (Desmedt et al., 2007), 1561 (Farmer et al., 2005), 2603 (Minn et al., 2005), 2990 (Sotiriou et al., 2006), GSE 9574 (Tripathi et al., 2008), GSE 15852 (Pau Ni et al., 2010), GSE 6883 (Liu et al., 2007) and 3494 (Miller et al., 2005). All experiments were performed following protocol GPL96, using total mRNA on the Affymetrix HGU133A microarray platform. This platform contains probes for 18,400 transcripts and variants. Raw microarray data was processed following a pipeline for *Robust Multi-array Average* (Irizarry et al., 2003), previously implemented in our workgroup (Baca-López et al., 2012; Tovar et al., 2015). Breast cancer samples were classified using the well-validated PAM50 algorithm (Parker et al., 2009).

Since all samples were downloaded from properly documented public databases, no ethics committee approval was required. All raw data is available in the NCBI-GEO database, with accession keys and references as stated above to guarantee full data availability.

### Sample comparability

Comparability is a key issue during analysis from microarrays, in particular when dealing with data coming from different sources (laboratories, technicians, etc.). Biases may exist even when the same protocols have been followed (Grass, 2009). Chen et al. (2011) tested six different algorithms to eliminate this so-called *batch effect* and found that the best results were obtained by using the empirical Bayesian assessment methods, such as **ComBat** (Johnson et al., 2007). The strategy followed here consisted in preprocessing all arrays with the **frma** algorithm (McCall et al., 2010), and using summarization with robust weighted average with no background correction, we split the datasets into cases/controls, and then applied **ComBat** to both datasets separately. After that, we re-joined the two resulting datasets and re-normalized them together with the **cyclic loess algorithm** (Ballman et al., 2004), in such way that both conditions belong now to the *same dynamic range.*

In order to assess the extent of this effect within our samples, so that we could remove the corresponding bias as accurately as possible, we resort to Principal Variance Component Analysis (PVCA) which is an algorithm combining the advantages of the principal component analysis (reduction of dimensionality) with the statistical reliability of the analysis of variance (Grass, 2009). After bias reduction, a PVCA analysis corroborated that such a confounding or batch effect almost disappeared. (Further information can be found in Supplementary Material 1).

### Transcriptional network inference

Gene regulatory network inference from experimental data involves the solution of an inverse problem (also called a deconvolution) which consists in unveiling the interactions (edges or links) from the properties of observables such as gene expression levels. Inferring a network implies the uncovering of the statistical dependencies within a Joint Probability Distribution (JPD). A usual way to do this is by quantifying the new information content that arise when we compare the full JPD to a series of successive independent approximations. In practice doing this is rather difficult because one is faced with large numbers of variables with a strong nonlinear behavior. Mutual Information (*MI*) is a measure from information theory that is able to deal with these issues since it is model independent, non-parametric and capable of capturing non-linear dependencies (Hernández-Lemus and Siqueiros-García, 2013).

Transcriptional network inference based on *MI* has been successfully employed (Hernández-Lemus and Siqueiros-García, 2013; Khosravi et al., 2015; Rodriguez-Barrueco et al., 2015). ARACNE (Margolin et al., 2006) is one of many algorithms used to calculate MI based on gene expression. ARACNE algorithm presents a relatively fast and reliable implementation of the inference of gene regulatory network from gene expression data. The method works as follows: a normalized gene expression matrix (i.e., an N by M matrix containing the gene expression levels of N genes in M samples) is used as input. The algorithm then calculates the *empirical* marginal probability distribution for the expression levels for all genes (i), as well as the *empirical* joint probability distribution for all the gene-couples (i,j) by approximating them by using Gaussian kernels. With these probabilities, a value of the *MI* between any two genes is calculated.

The method associates a *MI* value to each significance value (*p*-value) based on permutation analysis, as a function of the sample size. Therefore, a *MI* threshold (*MI*_0_) can be defined. For every pair of genes with *MI*_*i, j*_ > *MI*_0_ an interaction of weight *MI*_*i, j*_ is reported. Pairs of genes with *MI*_*i, j*_ < *MI*_0_ are considered to be non-interacting. Correlation analysis is made in the presence of *range restriction*, i.e., a sufficient amount of variance to allow for the detection of correlation structure. Hence a system observed at different scales (as given by the cut-off values) will give rise to different solutions. As it has long been known, in biological systems there is no *preferential scale of observation* a fact that makes scaling analysis a relevant approach to the quantitative analysis in biology (Giuliani et al., 2004).

Using the previously described expression data, we inferred transcriptional networks for each of the following molecular subtypes of breast cancer: Luminal A, Luminal B, Basal and HER2-enriched, and one for the non-tumor breast tissue phenotype. The construction of our networks proceeded as follows:

1. MI was calculated for every pair of (non-self) probesets in the microarray platform, using the ARACNE algorithm.
2. Those interactions ranked highest by Mutual Information values were kept.
3. Probesets were mapped to HUGO gene symbols. Those probesets that did not correspond to a gene symbol were discarded.

#### Network analysis and comparison

In order to compare the network structure of each phenotype, we analyzed each network by calculating the following metrics: the number of nodes and edges; the node degree (number of nodes connected to a specific node); connected components (a subset of nodes connected among them and not connected to the rest of nodes in the graph); and clustering coefficient (the number of existing node triplets over the total number of triplets) (Luce and Perry, 1949; Watts and Strogatz, 1998). Network connectivity degree is perhaps the most obvious *centrality measure*, i.e., it is an indicator of the relevance of a particular node to the large scale structure of the network. Genes that are more connected (that is, have a higher degree) are the ones that partake in most interactions, thus connecting a largest number of biological processes. Hence, highest degree genes are known to be quite important for the establishment of phenotypes.

The number of connected components (or islands) in a complex biological networks is a simple yet important indicator about the way in which different parts of the network (subnetworks) work together. A small number, or even a single connected component means that most (or all of the) interactions in the network have impact at a *global* level, whereas a higher number of connected components may imply a certain degree of *modularity* in which interactions within a subnetwork are somehow *autonomous* from interactions in other subnetworks.

The clustering coefficient in these networks is also indicative of the modularity and connectivity patterns at a lower (more local) scale than that of the number of connected components. Higher values of the average clustering coefficient can be related to greater redundancy and robustness in biological networks. Network topological analysis and visualization was performed by using Cytoscape v.3.0 (Shannon et al., 2003).

As previously mentioned, a network is defined by a set of nodes and a set of links between said nodes. Particularly in these inferred networks, nodes are representing genes and links correspond to potential transcriptional interactions. Similarity in these sets among phenotypes points to similarity in transcriptional programs. To compare these sets we used the **Jaccard index** *J*, this is obtained by dividing the size of the intersection over the size of the union of two sets. This was done for gene sets and link sets.

Jaccard indexes are used as measures of *similarity* between two sets: the closer *J* is to 1, the more similar the sets; Conversely, *J* = 0 implies completely dissimilar sets. Indeed, Jaccard indexes are actual probability measures. Here, Jaccard indexes are calculated for the sets of nodes (genes) of different networks, to see to what degree transcriptional networks representing different phenotypes (breast cancer subtypes) share genes, regardless of their particular interactions. On the other hand, Jaccard indexes calculated for the sets of interactions or links in those networks, reveal to what extent different transcriptional regulatory programmes share, not only groups of genes but also connection patterns among those genes.

Since biological functions depend not only in sets of specific molecules, but also in the interaction patterns among them, the joint consideration of similarities (and dissimilarities also) between gene sets (lists) and interaction sets may broaden our scope as to what are the differences and commonalities of breast cancer subtypes in terms of *biological features* (García-Campos et al., 2015).

#### Network threshold assessment

As already mentioned the cut-off value in the *MI* distribution will affect the membership of particular interactions as well as the structure of the inferred networks. In some sense, the choice of this threshold is indeed related to *feature selection*. The choice of cut-off value to construct *meaningful* (at the light of the feature selection procedure) networks is an open problem in contemporary research in biology. This is to say that, a particular cut-off choice depends on what kinds of features are to be selected (Giuliani et al., 2004; Censi et al., 2011). In order to avoid unnecessary biases, we decided to test different *MI* cut-off values compliant with quite general topological structure constraints of the underlying networks. Network size, for instance, is one of the most important constraints: extremely small and stringent networks will not capture the essential biological information, whilst extremely large, low confidence networks will present a larger number of false positive interactions and are much harder to analyze in order to unveil biological function. We assessed the threshold influence by calculating network metrics for different cut-off values, restricting our analysis to those networks which have a node-to-link ratio around 0.1, as this ratio value is characteristic of biological complex networks (Albert and Barabási, 2002; Barabasi and Oltvai, 2004; Barabási et al., 2011).

## Results

We inferred transcriptional networks for each breast cancer molecular subtype and for non-tumor mammary tissue. Network approaches are highly relevant for the understanding of the connection between sample state-variability and gene expression patterns, essential to elucidate the role that such expression patterns play in the establishment of cellular phenotypes. In this work we decided to pursue this by calculating mutual information correlation measures rather that parametric correlation coefficients due to the higher generality of the former.

Based on the evaluation of network threshold (Supplementary Material 2), we will present the results for networks constructed from the 10,000 interactions ranked with highest *MI* values, which are also, as previously mentioned, those with the highest statistical significance (*p-value* < 10^−10^). This cut-off value lies in the identified range (between the 10^3^ and 10^5^ highest ranked interactions) in which the node-to-link ratio is consistent with the expected values for biological complex networks.

### Transcriptional networks show different architectures

**Figure 1:**
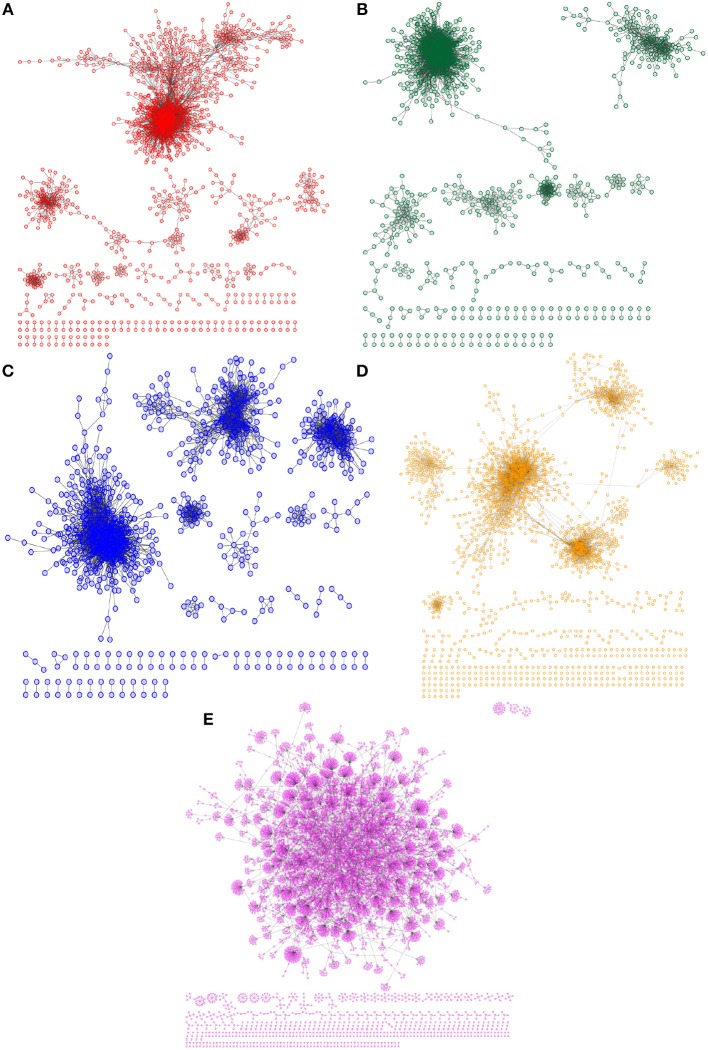
**Network architectures for breast cancer subtypes and non-tumor breast tissue**. In each panel, the transcriptional network structure of each breast tumor subtype is shown: **(A)** Luminal A (red nodes); **(B)** Luminal B (green); **(C)** Basal (blue) **(D)** and HER2-enriched molecular subtype (orange). **(E)** Shows the transcriptional architecture for non-tumor breast tissue. Please notice the that **(A–D)** show networks with a large component and multiple medium-sized components, while **(E)** presents a network dominated by a single giant component, followed by small components.

A graphical representation of all phenotype networks constructed with our approach can be observed in Figure 1, starting with the 4 breast cancer subtypes (Figures 1A–D), and the non-cancer mammary tissue network (Figure 1E). In this representation, nodes correspond to genes and links are a representation of the *MI* value. We will now show the main differences in the transcriptional network gene composition, highlighting the differences both between non-tumor and tumor networks as well as between tumor subtypes.

Figure 1 shows the differences in transcriptional network architectures among phenotypes. These differences in structure can be thought of as a representation of potential differences in the transcriptional regulatory program. As it has been discussed previously (Censi et al., 2011), in systems under stress (high correlation, and high variance) (Gorban et al., 2010) changes in the general correlation structure become visible as changes in the associated networks. The most evident differences in network structure are seen between the tumor and the non-tumor networks: the non-tumor network is dominated by a giant component; meanwhile, the subtype networks are formed by several coexisting components of different sizes. In this regard, we can expect that changes in the gene correlations between the normal and disease conditions may appear, at the local—pairwise correlations—, modular—or functional—and global—at the genome-wide transcriptional network—levels (Censi et al., 2011). It is also evident that while more similar among themselves, each breast cancer subtype has a unique network architecture. This is reflected in the network metrics reported in Table 1.

**Table 1:** **Network metrics**.

**Parameter**	**Luminal A**	**Luminal B**	**Basal**	**HER2-enriched**	**Non-tumor**
Nodes	1451	1018	1046	2100	1027
Edges	9941	9898	9966	9856	9894
Connected components	88	70	56	162	46
Clustering coefficient	0.3658	0.4044	0.4114	0.3586	0.3722
Min *P*-value	5.09 × 10^−25^	1.34 × 10^−23^	2.2 × 10^−21^	1.95 × 10^−12^	9.06 × 10^−19^

#### Non-tumor transcriptional network architecture

The transcriptional network for non-tumor tissue is shown in Figure 1E. This network is dominated by a giant component which contains 913 genes and 9823 links between them; in other words, about 90% of the genes in the network belong to this larger component, with the rest scattered in small islands of less than ten genes. The network's clustering coefficient (CC) is 0.3722, which indicates a sparse network structure. This particular network architecture, a *sparse network mostly comprised by a giant component*, was found to be unique to the non-tumor phenotype.

#### Breast cancer transcriptional networks show different topologies between molecular subtypes

The transcriptional networks for each molecular subtype are shown in Figures 1A–D. This graphical representation makes immediately evident the architectural differences between networks. Luminal A subtype (Figure 1A) shows a network with one larger component, four other mid-sized components with a small number of connected genes, and several small islands of less than 10 genes. This network contains 1451 annotated genes, with 9,941 links between them, and a CC of 0.3658.

The Luminal B subtype network (Figure 1B) shows one larger component, followed by four other smaller components, and several genes scattered in small islands of less than 20 genes. This network has a total of 1018 genes, with 9898 links between them, and a CC of 0.4044.

In the Basal subtype network (Figure 1C), we observe again a larger component, but in this network, the next two components in size are in the same order of magnitude, and smaller sized islands ranging from thirty to two nodes in size. This network has 1046 nodes and 9966 links between them, and a CC of 0.4114.

Finally, the network for the HER2-enriched subtype (1d) has an architecture dominated by a larger component, which is composed by several clusters linked together by few genes acting as bridges; the rest of the network is composed from islands ranging in size from 40 to 2 nodes. This network has the largest number of individual genes (2100) and 9856 links between them, with a CC of 0.3586.

It is important to notice that the disruption of connected components in the cancer networks is a phenomenon that it is very likely intertwined with the presence of stress induced correlations (Gorban et al., 2010), as such one need to be cautious as to assign a degree of importance to each of these two features on the issue of phenotypic differences on subtype-associated networks.

### Network composition analysis

Networks were constructed following restrictions that makes them comparable in size. However, it was found that each phenotype is has a unique network composition of both genes and links between them. Network composition constitutes a different level of analysis in the multi-scale characterization of biological systems (Giuliani et al., 2004). The actual molecular make-up of gene regulatory networks extends its influence not only to the upper level—the topological network structure itself—but also to the somewhat lower scale level of description given by the molecular pathways and particular biochemical processes behind physiological functions and phenotypes (Censi et al., 2011), for this reason, in what follows we will present a detailed description of the molecular composition for phenotype (i.e., subtype)-specific networks.

#### Transcriptional networks reveal different gene compositions

**Figure 2:**
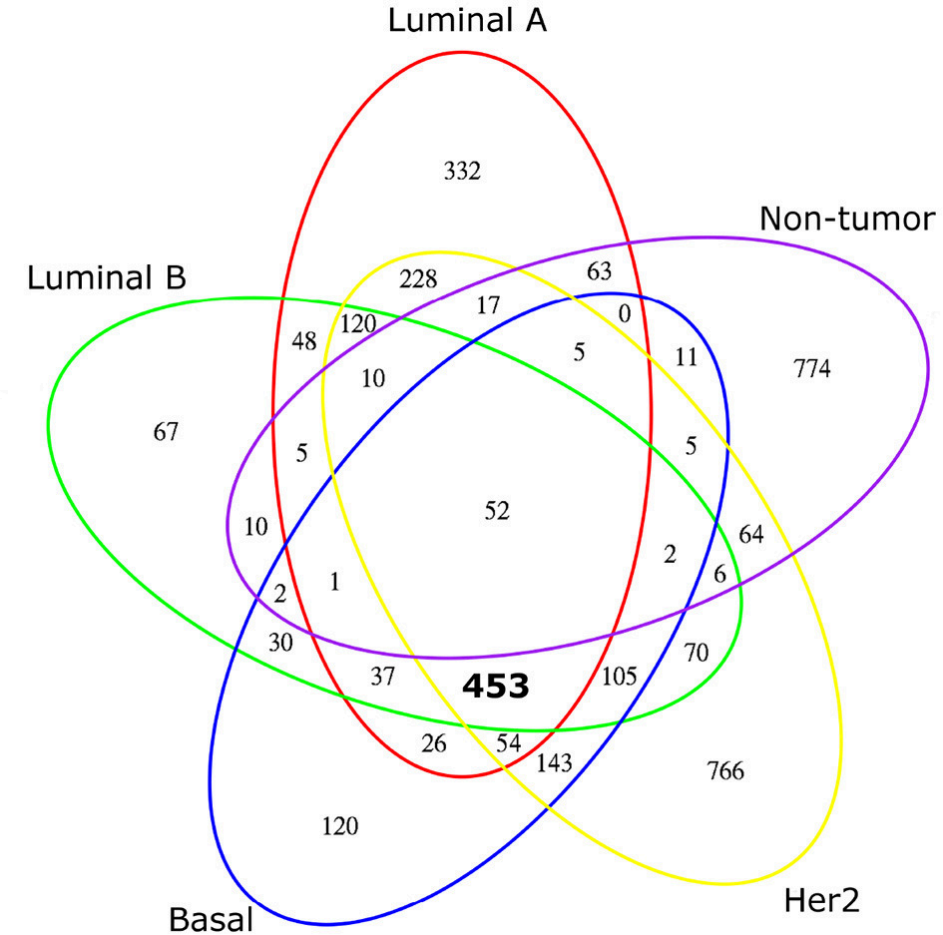
**Venn diagram of network node composition**. A five-set Venn diagram of the nodes that form each transcriptional network: Luminal A is outlined in red, Luminal B in green, Basal in blue, HER2-enriched in yellow and non-tumor breast tissue in purple. Numbers in graph represent how many genes belong to each subset. Notice that the intersection of the four breast cancer molecular subtypes is large (453, marked in bold); meanwhile the number of shared genes between tumor and non-tumor networks is much smaller, with only 52 genes shared by all networks.

Each transcriptional network inferred contains a particular set of genes. Each of these sets is not completely dissimilar to another, as there are intersections between them. Figure 2 shows a Venn diagram representation of the overlap between gene sets for each transcriptional network. In this figure, it can be seen that the intersection of genes in all breast cancer and non-cancer transcriptional networks is relatively small, containing only 52 genes. Meanwhile, the intersection of genes in all breast cancer networks, excluding those shared with the non-cancer network, contains 453 genes, a “breast cancer core set” which we will discuss later. Finally, notice how the number of non-shared, “exclusive” genes for each breast cancer transcriptional network is different.

The differences between network genesets can also be seen in Table 2, containing *J* for each network pair. This table shows varying levels of similarity between molecular subtypes: we can see how the Luminal A and Basal subtypes are the most different in gene composition, and interestingly, the most similar in gene composition are the Luminal B and Basal subtypes.

**Table 2:** **Jaccard indexes for each pair of transcriptional network node sets**.

	**Luminal A**	**Luminal B**	**Basal**	**HER2-enriched**	**Non-tumor**
Luminal A	–	0.4165	0.3360	0.3595	0.0658
Luminal B	0.4165	–	0.4935	0.3556	0.0450
Basal	0.3360	0.4935	–	0.3519	0.0391
HER2-enriched	0.3595	0.3556	0.3519	–	0.0543

Even more striking is the fact that similarity between any cancer network nodeset and the non-tumor network nodeset is about an order of magnitude smaller than any *J* value for any cancer network pair, indicating that non-tumor network vastly differs from any breast cancer network; with 774 unique genes, the non-tumor transcriptional network is 90% unique.

#### Transcriptional networks reveal different gene-interaction compositions

Transcriptional networks show even more differences in their link composition. Table 3 shows *J* values indicating the similarity in link sets between networks. Again, it is interesting to note that the highest similarity in links is between the Luminal B and the Basal networks, followed by the similarity between the Luminal A and Luminal B networks. Again, the linkset similarity between non-tumor and any tumor network, is about two orders of magnitude smaller than between any two tumor networks.

**Table 3:** **Jaccard indexes for each pair of transcriptional network link sets**.

	**Luminal A**	**Luminal B**	**Basal**	**HER2**	**Non-tumor**
Luminal A	–	0.2181	0.1461	0.0956	0.0024
Luminal B	0.2181	–	0.2254	0.1176	0.0014
Basal	0.1461	0.2254	–	0.1189	0.0014
HER2	0.0956	0.1176	0.1189	–	0.0014

The differences (and similarities) in link sets are determinant for phenotype definition. Even though gene composition describes *which molecules participate* in the transcriptional landscape, the *potential transcriptional interactions* that exist between each other, are the essence of the regulatory program.

#### A core set of genes is shared among breast cancer network subtypes

**Figure 3:**
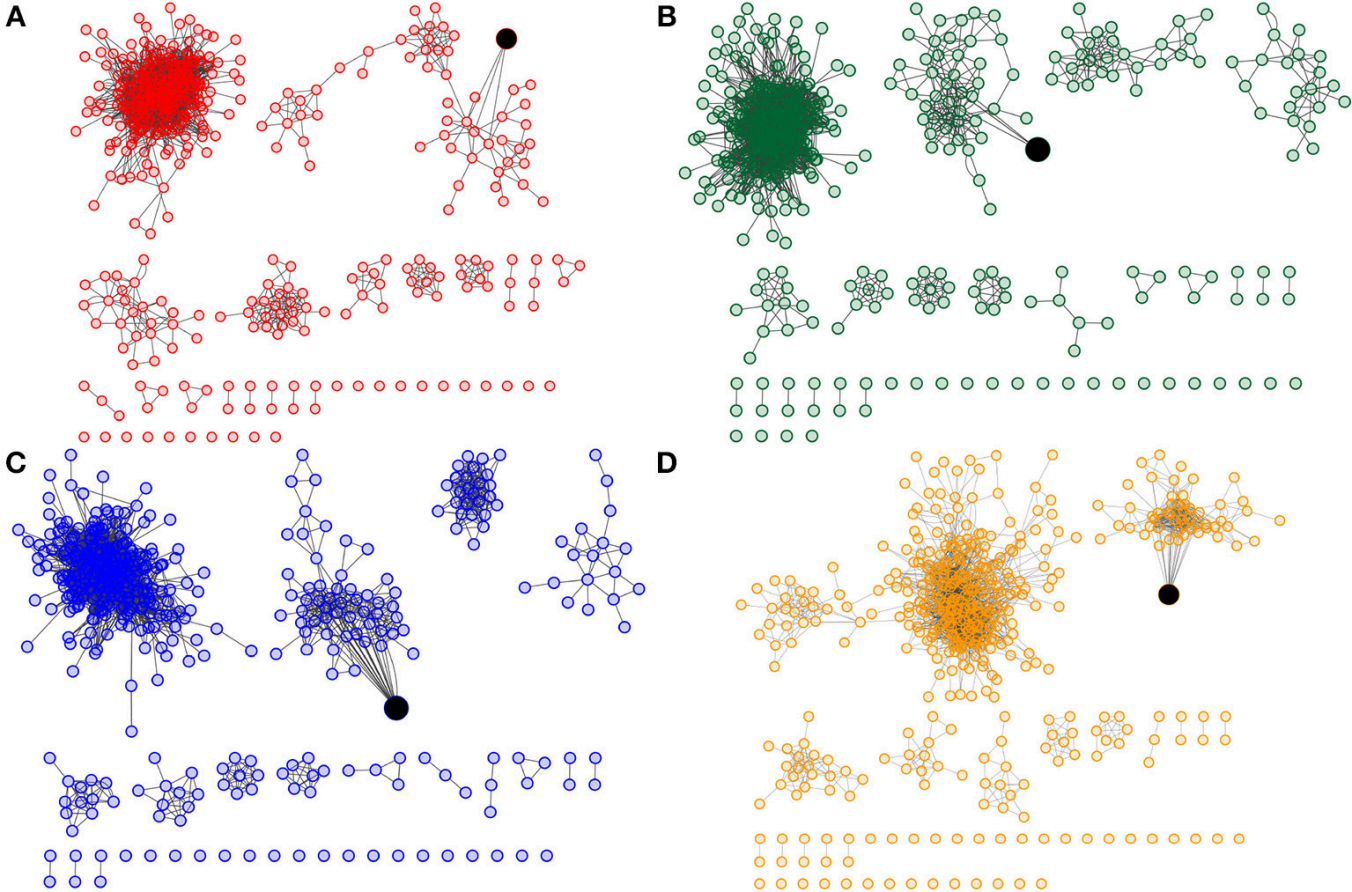
**Subnetworks of *core genes* shared by all breast cancer networks have different connections**. **(A–D)** Show the 453 core genes for Luminal A, Luminal B, Basal and HER2-enriched subtypes. All subnetworks have a component with IRF8 transcription factor (shown in black).

As we have shown before, both gene and link composition define distinct network architectures for each transcriptional network, reflecting the heterogeneous nature of cancer. Particularly, different connections between the same genes may be involved in the development of features specific to each subtype. This can be evident, for instance, in the connections between the previously described *breast cancer core set* of genes shared by all breast cancer networks. Figure 3 shows the subgraphs of each breast cancer network containing only these core genes. These highlight how, even when taking into account the same genes, the interactions between them vary across the molecular subtype landscape. As an example, take the isolated component which contains the IRF8 transcription factor, present in all subnetworks; notice how the degree of IRF8 is different in all subtypes: ranging from only three neighbors in the Luminal A subtype, to 21 neighbors in the HER2-enriched subtype.

### Node degree as a measure of relative influence of genes in the transcriptional context

We have focused on global network properties which define breast cancer network structures. In any network, each node has individual topological properties related to the global structure. For instance node degree, the number of neighbors of a particular node, is a measure of its centrality in the network context. For our purposes, we can think of node degree in breast cancer transcriptional networks as a measure of the influence which a particular gene may have in the regulatory program. In this sense, transcriptional network architecture not only gives global information about phenotypes, but also insights on the role of particular genes.

**Figure 4:**
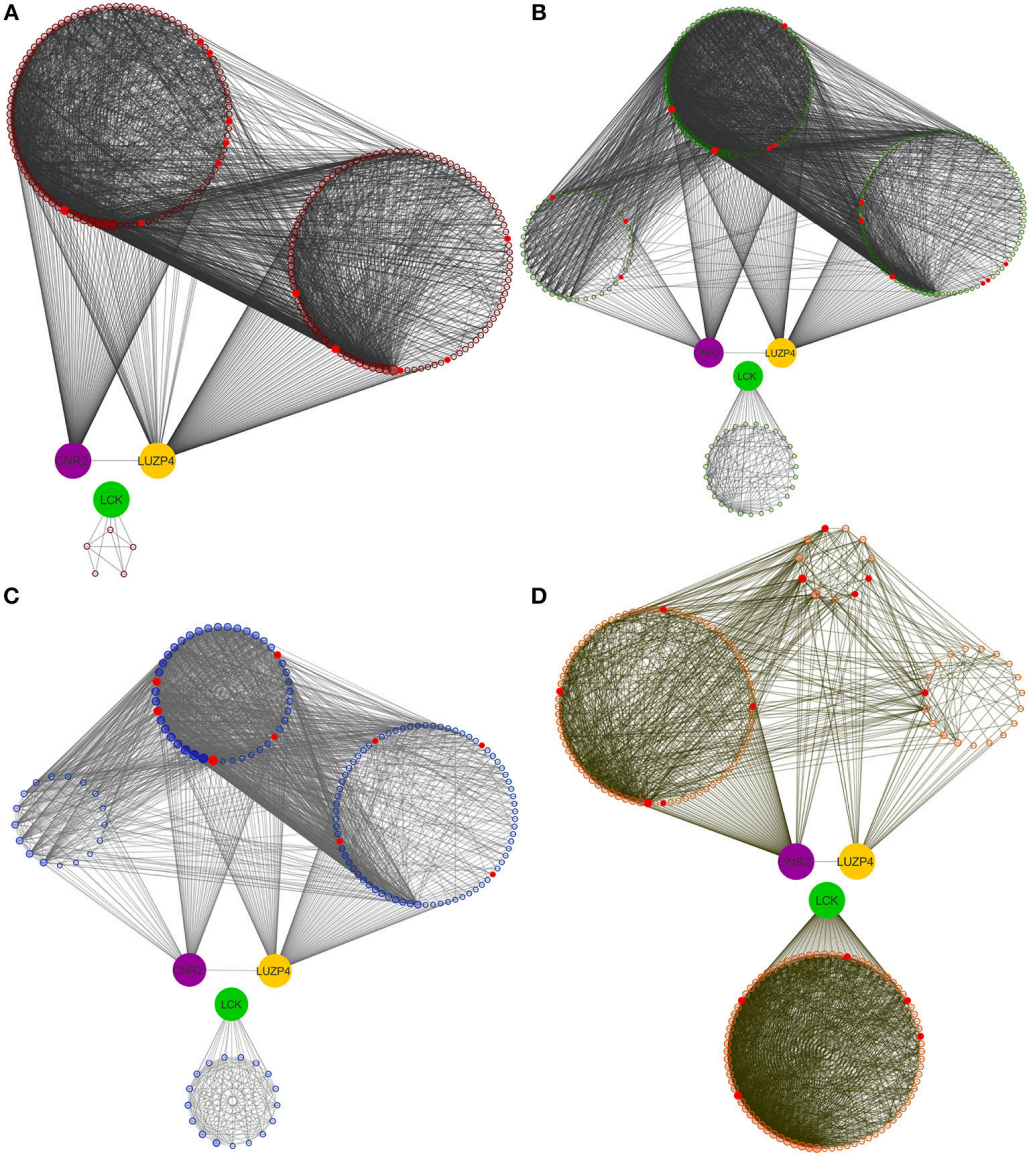
**First neighbors of CNR2, LCK, and LUZP4 genes**. Genes that are connected to CNR2 (violet), LCK (green) and LUZP4 (yellow) are shown. **(A–D)** Show the same order than Figures 1, 2. It can be seen that CNR2 is highly connected in all subtypes; LCK gene has a high degree relevance in HER2-enriched subtype. Finally, LUZP4 gene has different degree relevance depending on the molecular subtype. It is worth noticing that CNR2 and LUZP4 share first neighbors, but not with LCK.

Given the differences in network structures, it can be expected that even the same genes may have different influences in each specific molecular subtype; at the same time, it is not unreasonable to think that some genes will play an important role in all molecular subtypes. As a proof of context, we have selected three genes which cover these scenarios: CNR2, LCK, and LUZP4. Those genes were selected on the basis of their being highly central in at least one of the cancer subtypes and being present in all the subtypes' networks, so as to have a rational means for comparison of the different effects they may have in different biological networks structures. Subnetworks containing these genes and their first neighbors for each breast cancer molecular subtype can be seen in Figure 4.

## Discussion

Deconvoluting transcriptional network architecture is a step toward uncovering differences between health and disease, as well as between different manifestations of disease. Doing this is relevant for two main lines of research: basic biological knowledge and clinical therapeutical interventions. On the one hand, unveiling the hidden relationship between system's level transcriptional programmes—as sketched by gene regulatory networks—and molecular and physiological phenotypes—given by the different breast cancer subtypes—is of great importance on the way to understand to a deeper level the elusive genotype/phenotype relationships (Barabási et al., 2011; Censi et al., 2011; Ahmad et al., 2012). On the other hand, detailed and specific knowledge about the differences and similarities of phenotype-specific gene regulatory networks, and their underlying biochemical processes as represented by metabolic and signaling pathways, may pave the way to find specific pharmacological targets useful in the clinical and therapeutical management of different breast cancer subtypes.

In this work, by using a computational approach based on information theory, we inferred transcriptional networks from genome-wide gene expression microarrays of the following breast cancer subtypes: Luminal A, Luminal B, Basal, and HER2-enriched; these were contrasted with comparable non-tumor breast tissue networks. We showed that the architecture of these transcriptional networks is unique to each phenotype, and emphasize the major differences between non-tumor and cancer networks, as well as the differences between different subtypes of breast cancer. We propose that these transcriptional networks reflect the transcriptional programs behind each phenotype, which is involved in the physiopathological differences observed in breast cancer. To our knowledge, this is the first time that transcriptional network structure has been characterized at any level of description for these clinically important breast cancer subtypes.

Breast cancer heterogeneity is a widely acknowledged fact, with molecular, histological, and clinical manifestations. In this work, we have shown breast cancer heterogeneity manifested in transcriptional network architectures; since molecular subtypes are defined by differences in gene expression profiles (see Supplementary Material 2), we could expect that transcriptional networks, derived from such data, would reflect this. It is well-known that network structure is intimately linked to functionality in biological networks (Kitano, 2002; Albert, 2005; Serrano, 2007; Hakes et al., 2008). In this case, we propose that our inferred networks are a representation of an underlying transcriptional program associated to each of the studied phenotypes.

We expected a drastic difference between non-tumor and tumor *networks*, just as there is a drastic difference between non-tumor and tumor *tissue*. Indeed, we found a marked difference in structure between breast cancer networks and a non-cancer network; this is in agreement with the evident differences in gene expression profiles between these two states (see Supplementary Material 2). The non-tumor transcriptional network is dominated by a giant component, while the networks for each of the breast cancer subtypes studied present a larger number of disconnected components. This suggests the existence of generalized transcriptional communication in healthy cells, which is lost and supplanted with a fractured, more autonomous regulation in different cancer manifestations. We consider that more research is needed in order to find mechanistic causes to this phenomenon.

The differences between cancer and non-cancer networks are also seen in the genetic composition of these networks, suggesting differences in the importance of genes in the regulatory programs of health and disease. There are only 52 genes which are part of both the non-tumor and all the cancer networks. Most of these are located in small “islands” in the network, where they interact with few other genes, rendering their impact to the global network topology negligible. Only 5 out of these 52 genes belong to the larger network components. These genes seem to be associated to general biological functions:

- ASCL3 encodes a transcription factor highly involved in determination of cell fate and the development and differentiation of numerous tissues (Yoshida et al., 2001; Jonsson et al., 2004).
- DNAJ4 encodes a highly conserved heat shock protein which serves a chaperone (Walker et al., 2010; Hageman et al., 2011).
- NCR1 encodes the natural cytotoxicity triggering receptor recognizes a broad spectrum of ligands in natural killer cells (Kruse et al., 2013), and has been observed to participate regulating several functions in those cells (Pembroke et al., 2014; Fu et al., 2015; Tanimine et al., 2016).
- SLN encodes sarcolipin, a Ca^2+^-ATPase located at the sarcoplasmic reticulum, catalyzes the ATP-dependent transport of calcium ion from the cytosol into the sarcoplasmic reticulum in muscle cells (Fajardo et al., 2013; Gorski et al., 2013; Espinoza-Fonseca et al., 2015).
- BMP15 encodes a protein associated to oocyte maturation, and follicular development (Fenwick et al., 2013; Persani et al., 2014; Sutton-McDowall et al., 2015).

Breast cancer heterogeneity, as reflected by molecular subtypes, is mostly related to the cellular composition of the normal mammary tissue: the mammary epithelium has an inner layer composed by luminal cells, and an outer layer formed by basal cells (Skibinski and Kuperwasser, 2015); the luminal and basal subtypes show phenotypical similarities to these cells. HER2-enriched breast cancer is mostly determined by an overexpression of the ERBB2 receptor, regardless of whether its cell exhibits luminal or basal characteristics. With this in mind, we expected transcriptional networks to reflect this. It would be expected to find common features in transcriptional networks for the two luminal subtypes, and also to think that these networks would be somewhat different from the basal network, with the HER2-enriched network perhaps exhibiting commonalities with all networks. Our results show the expected similarity between the luminal A and B transcriptional networks. However, it was surprising to find that the highest similarity in both nodes and links was not between them, but rather between the luminal B and Basal networks. Meanwhile, the HER2-enriched subtype network showed was found to have the most unique architecture, as well as being the most different of all breast cancer networks. It is worth noting that we were able to recover a genetic network composition that captures the differences between normal breast and breast cancer tissue, as well as the differences between the various molecular subtypes, at the gene expression level (see Supplementary Material 2).

We believe that these results suggest that the oncogenic processes behind breast cancer originate unique transcriptional programs which drive each molecular subtype. The similarities of the luminal B subtype network to both the luminal A and the Basal networks are in line with the idea of a common breast cancer progenitor cell for these subtypes; at the same time, the nature of the HER2-enriched network could be indicative of a different molecular origin. (Skibinski and Kuperwasser, 2015).

The existence of a “core set” of genes which are shared among all breast cancer subtype networks was noteworthy. More interesting was the fact that even though all subtypes share these genes, the wiring structure of these is not the same in each phenotype, which again suggests that differences in the regulatory program are driven not only by the genes that participate, but by the relationships between them. The transcriptional networks define a landscape in which different elements may be playing distinct roles. Therefore, our breast cancer transcriptional networks not only provide us with global network features, but are also useful in order to identify the influence that any given gene may have in the transcriptional program. In order to illustrate how the same genes may have different roles in different breast cancer manifestations, we selected, based on their degree in each breast cancer network, the CNR2, LCK, and LUZP4 genes.

CNR2 is a gene highly connected in all breast cancer networks. CNR2 codifies the cannabinoid receptor 2 (CB2). This receptor is associated to immunomodulation and related processes by endocannabinoids (Munro et al., 1993). CB2 alterations have been found in different types of cancer (Guida et al., 2010; Jha et al., 2012; Pisanti et al., 2013), including breast cancer (Nasser et al., 2011; Pérez-Gómez et al., 2015; Sophocleous et al., 2015). Our analysis identifies CNR2 as one of the top 30 highest degree nodes in our breast cancer subtype transcriptional networks. Based on this, CNR2 might be pointed as an important gene in the general breast cancer transcriptional architecture. Cannabinoid receptors have been previously proposed as pharmacological targets for cancer (Chakravarti et al., 2014; Velasco et al., 2016), including breast cancer (Qamri et al., 2009; Morales et al., 2015). Our transcriptional network findings suggest that, if therapeutic benefits to this type of treatment are found, they may be of use to all types of breast cancer.

LCK gene was identified as a key player in HER2-enriched subtype while having a low degree in the other molecular subtypes network. LCK codifies the LCK proto-oncogene, Src family tyrosine kinase, a protein involved in signal transduction. LCK has been found expressed in breast cancer (Köster et al., 1990). Furthermore, a role in breast cancer progression and angiogenesis has been identified (Chakraborty et al., 2006). Interestingly enough, there are reports of a LCK-associated molecular signature with prognostic utility in HER2-enriched breast tumors (Rody et al., 2009). The result presented here reinforces the functional implication of LCK in the context of HER2-enriched breast cancer and emphasizes the necessity of further, focused studies.

LUZP4 was identified as a high degree gene in all breast cancer networks, except in the HER2-enriched. LUZP4 codifies a leucine zipper protein. This protein has not been extensively explored, however, recently, this leucine zipper has been identified as involved in mRNA exporting in cancer cells (Viphakone et al., 2015). Our results indicate that this gene may be an important player in Luminal A, Luminal B, or Basal. Furthermore, unlike the two previously discussed genes, LUZP4 has not been extensively studied in the context of breast cancer. This gene is an example of the type of new biological information that can be recovered from existing data only with the use of network-based approaches. We believe that further experimental exploration of this molecule may be of interest in the future.

As it has been largely discussed (Margolin et al., 2006; Baca-López et al., 2012), the probabilistic inference of gene regulatory networks is fundamental (and in some cases almost mandatory) to unveil complex transcriptional interactions that would be otherwise extremely difficult to notice. However, as it has also been noticed (Margolin et al., 2006), reverse engineering methods have also a number of limitations. In the particular case of transcriptional networks inferred via Mutual Information calculations, one important aspect is that regulatory interactions may include not only canonical transcription factor to target interactions, but also a number of indirect and more complex relationships. In this sense, MI-inferred networks (such as the ones discussed here) provide us with information about statistical dependency in the transcriptional profiles (Hernández-Lemus et al., 2009; Baca-López et al., 2012; Creixell et al., 2015).

A major open question in the field of network reconstruction is still how to establish a proper cut-off value to include a link in a network (Serrano et al., 2009). We decided to make this choice based on two constraints: on one hand, to include links with a high statistical confidence, as measured with the associated *p*-value provided by ARACNE, and on the other one, to obtain a network that belongs to the *complex network regime*, as described by Albert and Barabási (2002), which exhibit a node-to-link ratio in which *nodes* << *links*.

It is evident that the threshold choice will modify network parameters. We identified that, in an interval surrounding our selected node-to-link ratio (0.1), network parameters are generally stable (Supplementary Material 3). More importantly, even though the actual numeric values may be modified, is important to notice that the behaviors such as similarity between networks (Supplementary Material 4) and the relative influence of genes, based on their node degree (Supplementary Material 5) are preserved in this interval. Importantly, this shows robustness of the transcriptional regulatory program, while at the same time allowing us to observe structural differences between phenotypes along the interval. It is worth noticing that the network structures obtained with our methodology are quite different to networks generated at random (see Supplementary Material 6).

We have shown how a network approach can be useful to understand the heterogeneity of breast cancer. We were able to infer and compare the transcriptional programs of breast cancer molecular subtypes, and contrast them with that of healthy mammary tissue. We showed how this paradigm can help to identify novel roles of molecules in different manifestations of breast cancer, with potential biomedical implications.

## Author contributions

GdA devised the experiment, implemented the methodologies and provided this manuscript's first draft. TV contributed to the discussion and visualization of results and helped in the revision of the manuscript. JE contributed to the analysis of results, discussed biological implications, and helped in the writing of the manuscript. EH proposed the experiment, participated in its design and coordination and helped in the writing of the manuscript. All authors read and approved the final manuscript.

### Conflict of interest statement

The authors declare that the research was conducted in the absence of any commercial or financial relationships that could be construed as a potential conflict of interest.

## Supplementary material

Supplementary Material 1 **Normalization of microarrays with batch effect correction and information on subtype composition of each microarray experiment**.

Supplementary Material 2 **Principal component analysis visualizations of gene expression**.

Supplementary Material 3 **A table containing the results of the network analysis performed for each phenotype at different cut-off values**.

Supplementary Material 4 **A table containing the node and edge Jaccard indexes *J* between each pair of phenotypes at different cut-off values**.

Supplementary Material 5 **A zipped file containing lists with the 100 highest ranked genes by degree for each phenotype at different cut-off values**.

Supplementary Material 6 **Null model for network connectivity: nodes from transcriptional networks were reconnected according to the Erdős-Rényi model**.
