# Supplementary material for: Transcriptional Network Architecture of Breast Cancer Molecular Subtypes
Source: Front Physiol. 2016 Nov 22;7:568. doi: 10.3389/fphys.2016.00568 (PMC5118907; doi:10.3389/fphys.2016.00568)
Supplement: Supplementary Material 4 — A table containing the node and edge Jaccard indexes J between each pair of phenotypes at different cut-off values. [file DataSheet4.pdf]

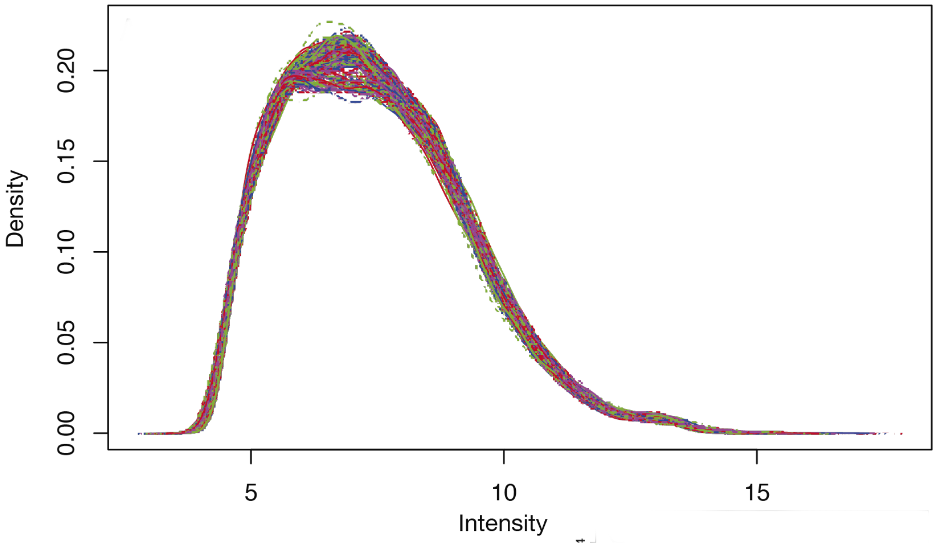

Figure 1: *

Batch effect control. Density plot of the GEO expression matrix used for this analysis after application of ComBat for cases and controls separately. Thereafter, samples were joined with a cyclic loess normalization.

Table 1: GEO identifier and references for the microarray experiments used here, the first column is GEO key ID, the second and third columns are the corresponding number of samples cases/controls, respectively. The fourth column is a brief description of the samples, and the fifth one presents the associated reference.

GEO ID Series	Luminal A	Luminal B	Basal	HER2-enriched	non-tumor	Reference
GSE1456	41	36	16	13	0	[7]
GSE1561	1	14	14	6	0	[2]
GSE2603	9	20	28	9	0	[5]
GSE2990	13	11	7	5	0	[8]
GSE4922	77	20	19	15	0	[3]
GSE7390	31	40	39	9	0	[1]
GSE6883	0	0	0	0	3	[4]
GSE9574	0	0	0	0	15	[9]
GSE15852 0	0	0	0	0	43	[6]

References

[1] Christine Desmedt, Fanny Piette, Sherene Loi, Yixin Wang, Françoise Lallemand, Benjamin Haibe-Kains, Giuseppe Viale, Mauro Delorenzi, Yi Zhang, Mahasti Saghatchian d'Assignies, Jonas Bergh, Rosette Lidereau, Paul Ellis, Adrian L Harris, Jan G M Klijn, John A Foekens, Fatima Cardoso, Martine J Piccart, Marc Buyse, Christos Sotiriou, and TRANSBIG Consortium. Strong time dependence of the 76-gene prognostic signature for node-negative breast cancer patients in the TRANSBIG multicenter independent validation series. *Clinical cancer research : an official journal of the American Association for Cancer Research*, 13(11):3207–3214, June 2007.

[2] Pierre Farmer, Herve Bonnefoi, Veronique Becette, Michele Tubiana-Hulin, Pierre Fumoleau, Denis Larsimont, Gaetan Macgrogan, Jonas Bergh, David Cameron, Darlene Goldstein, Stephan Duss, Anne-Laure Nicoulaz, Cathrin Brisken, Maryse Fiche, Mauro Delorenzi, and Richard Iggo. Identification of molecular apocrine breast tumours by microarray analysis. *Oncogene*, 24(29):4660–4671, July 2005.

[3] Anna V Ivshina, Joshy George, Oleg Senko, Benjamin Mow, Thomas C Putti, Johanna Smeds, Thomas Lindahl, Yudi Pawitan, Per Hall, Hans Nordgren, John E L Wong, Edison T Liu, Jonas Bergh, Vladimir A Kuznetsov, and Lance D Miller. Genetic reclassification of histologic grade delineates new clinical subtypes of breast cancer. *Cancer research*, 66(21):10292–10301, November 2006.

[4] Rui Liu, Xinhao Wang, Grace Y Chen, Piero Dalerba, Austin Gurney, Timothy Hoey, Gavin Sherlock, John Lewicki, Kerby Shedden, and Michael F Clarke. The prognostic role of a gene signature from tumorigenic breast-cancer cells. *The New England journal of medicine*, 356(3):217–226, January 2007.

[5] Andy J Minn, Gaorav P Gupta, Peter M Siegel, Paula D Bos, Weiping Shu, Dilip D Giri, Agnes Viale, Adam B Olshen, William L Gerald, and Joan Massagué. Genes that mediate breast cancer metastasis to lung. *Nature*, 436(7050):518–524, July 2005.

[6] Ivyna Bong Pau Ni, Zubaidah Zakaria, Rohaizak Muhammad, Norlia Abdullah, Naqiyah Ibrahim, Nor Aina Emran, Nor Hisham Abdullah, and Sharifah Noor Akmal Syed Hussain. Gene expression patterns distinguish breast carcinomas from normal breast tissues: the Malaysian context. *Pathology, research and practice*, 206(4):223–228, April 2010.

[7] Yudi Pawitan, Judith Bjöhle, Lukas Amler, Anna-Lena Borg, Suzanne Egyhazi, Per Hall, Xia Han, Lars Holmberg, Fei Huang, Sigrid Klaar, Edison T Liu, Lance Miller, Hans Nordgren, Alexander Ploner, Kerstin Sandelin, Peter M Shaw, Johanna Smeds, Lambert Skoog, Sara Wedrén, and Jonas Bergh. Gene expression profiling spares early breast cancer patients from adjuvant therapy: derived and validated in two population-based cohorts. *Breast cancer research : BCR*, 7(6):R953–64, 2005.

[8] Christos Sotiriou, Pratyaksha Wirapati, Sherene Loi, Adrian Harris, Steve Fox, Johanna Smeds, Hans Nordgren, Pierre Farmer, Viviane Praz, Benjamin Haibe-Kains, Christine Desmedt, Denis Larsimont, Fatima Cardoso, Hans Peterse, Dimitry Nuyten, Marc Buyse, Marc J Van de Vijver, Jonas Bergh, Martine Piccart, and Mauro Delorenzi. Gene expression profiling in breast cancer: understanding the molecular basis of histologic grade to improve prognosis. *Journal of the National Cancer Institute*, 98(4):262–272, February 2006.

[9] Anusri Tripathi, Chialin King, Antonio de la Morenas, Victoria Kristina Perry, Bohdana Burke, Gregory A Antoine, Erwin F Hirsch, Maureen Kavanah, Jane Mendez, Michael Stone, Norman P Gerry, Marc E Lenburg, and Carol L Rosenberg. Gene expression abnormalities in histologically normal breast epithelium of breast cancer patients. *International journal of cancer. Journal international du cancer*, 122(7):1557–1566, April 2008.
