# Supplementary material for: Transcriptional Network Architecture of Breast Cancer Molecular Subtypes
Source: Front Physiol. 2016 Nov 22;7:568. doi: 10.3389/fphys.2016.00568 (PMC5118907; doi:10.3389/fphys.2016.00568)
Supplement: Supplementary Material 5 — A zipped file containing lists with the 100 highest ranked genes by degree for each phenotype at different cut-off values. [file DataSheet5.pdf]

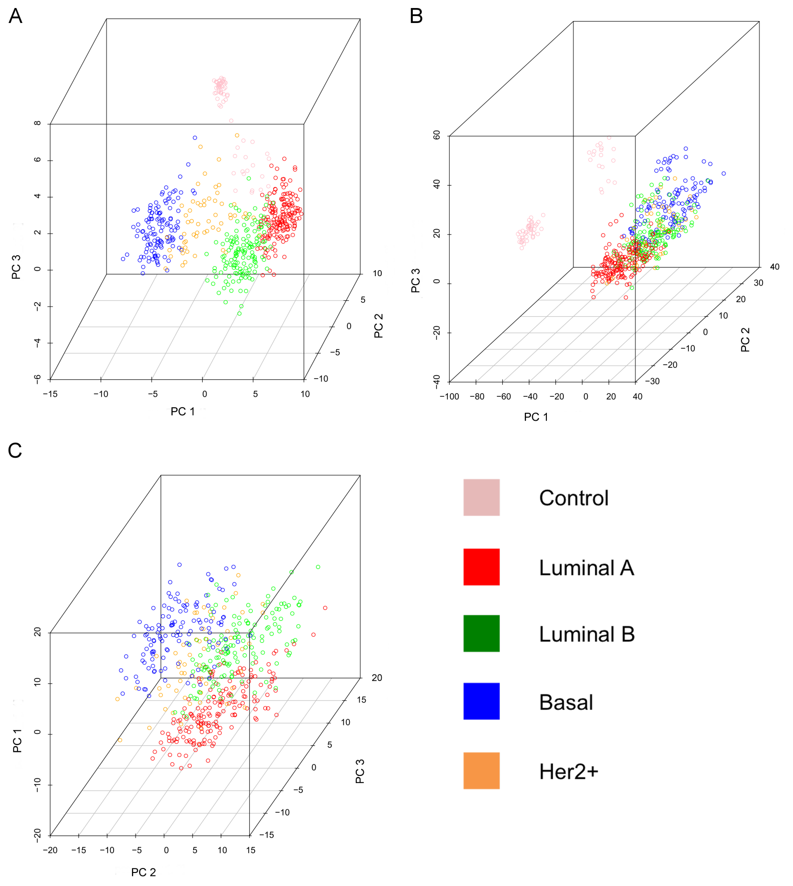

Figure 1. Principal Component Visualization of gene expression. In each panel, points represent microarray samples, color-coded by phenotype. Each axis represents a principal component obtained from a matrix where each sample is described by the expression levels of the following genesets: A) PAM50 genes B) Genes of tumor and non-tumor breast tissue networks, C) The combined set of genes unique to each of the four breast cancer molecular subtype networks. It can be observed the sample separation between the five phenotypes by using the PAM50 geneset. Meanwhile, the geneset corresponding to all genes present in every network analyzed is able to appropriately separate tumor from non-tumor samples. Finally, the genes that are unique to each of the breast cancer networks allow an adequate separation of the four molecular subtypes of breast cancer.
