# Supplementary material for: Transcriptional Network Architecture of Breast Cancer Molecular Subtypes
Source: Front Physiol. 2016 Nov 22;7:568. doi: 10.3389/fphys.2016.00568 (PMC5118907; doi:10.3389/fphys.2016.00568)
Supplement: Supplementary Material 6 — Null model for network connectivity: nodes from transcriptional networks were reconnected according to the Erdős-Rényi model. [file DataSheet6.pdf]

Table 1: Network metrics of the null model. The network construction was performed by recconnecting nodes according to the Erdös-Renyi model

Parameter	Luminal A	Luminal B	Basal	HER2-enriched	Non-tumor
Nodes	1,451	1,018	1,046	2,100	1,027
Edges	10,013	9,880	9,972	9,827	9,966
Connected components	1	1	1	2	1
Clustering Coefficient	0.0100	0.0190	0.0184	0.0041	0.0189

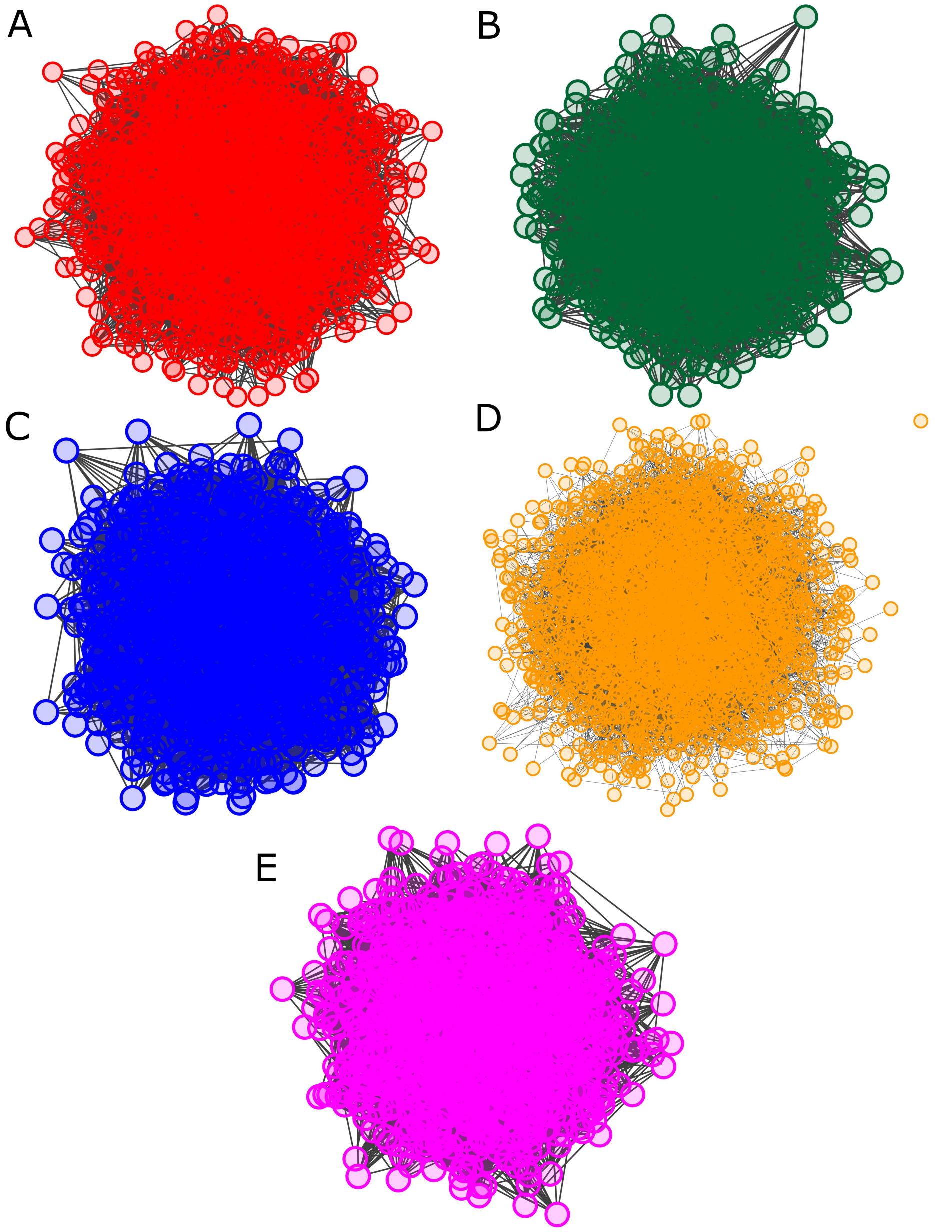

Figure 1. Visualization of the null model networks. The network construction was performed by recconnecting nodes of each phenotype network (luminal A, luminal B, basal, HER2-enriched, and non-tumor) according to the Erdös-Renyi model.
